# Preparation of a Low Reducing Effect Sulfonated Alkali Lignin and Application as Dye Dispersant

**DOI:** 10.3390/polym10090982

**Published:** 2018-09-03

**Authors:** Yanlin Qin, Xuliang Lin, Yaoqin Lu, Siyuan Wu, Dongjie Yang, Xueqing Qiu, Yanxiong Fang, Tiejun Wang

**Affiliations:** 1 School of Chemical Engineering and Light Industry, Guangdong University of Technology, Guangzhou 510006, China; ylqin@gdut.edu.cn (Y.Q.); xllin@gdut.edu.cn (X.L.); lyqgduthc@163.com (Y.L.); wusiyuan1201@163.com (S.W.); 2 Guangdong Provincial Key Lab of Green Chemical Product Technology, Guangzhou 510000, China; cedjyang@scut.edu.cn (D.Y.); cexqqiug@scut.edu.cn (X.Q.); 3 School of Chemistry and Chemical Engineering, South China University of Technology, Guangzhou 510000, China; 4 State Key Lab of Pulp and Paper Engineering, South China University of Technology, Guangzhou 510640, China

**Keywords:** alkali lignin, hydroxypropyl sulfonation, blocking condensed, reducing effect, dye exhaustion

## Abstract

A novel grafting hydroxypropyl sulfonated and blocking condensed lignin (GSBAL) dye dispersant was prepared based on alkali lignin (AL) by sulfonation and etherification reactions. The significant increase in the sulfonic group content and the molecular weight endow GSBAL with excellent dispersity and stability at high temperatures. More importantly, the unfavorable property of the reducing effect of AL was largely reduced since over 80% of the phenolic hydroxyl groups were blocked. The functional azo groups in the dye could be mostly retained. The reducing rate of dye with GSBAL was decreased to 6.54% (25 °C), much lower than 18.62% for sulfomethylated alkali lignin (SAL) and 15.73% for sodium lignosulfonate (NaLS). The dispersity and exhaustion of the dye bath with GSBAL dispersant was significantly improved compared with that of a dye bath with SAL and NaLS.

## 1. Introduction

The textile industry is the largest consumer of disperse dye. Azo dyes contain at least one azo group and account for approximately 70% of disperse dyes [1]. A disperse dye with poor water solubility cannot be diffused into polyester fiber efficiently [2]. Hence, it is necessary to add dispersant to dye to increase its dispersion, stability, and reduce particle size to promote dyeing efficiency. A typical dosage of dispersant ranges from 75% to 200% of dried dye cake mass. The most commonly used dispersants for water-insoluble dyes (most of them are not azo dyes) are sulfonated lignin and naphthalene sulfonates. However, the applications of naphthalene sulfonates are limited due to its high price and toxicity [3].

Two kinds of industrial lignin are byproducts in the papermaking industry. One is lignosulfonates (LS), which come from the sulfite-pulping process and contain water-soluble sulfonic groups. Another is alkali lignin (AL), which comes from the alkali pulping process at elevated temperatures (170 °C). AL is only dissolved in alkali solution due to a lack of hydrophilic groups. Sulfonation is the most effective way to improve the water solubility of AL and thus, broaden its applications. Typically, AL was converted into a soluble derivative named sulfomethylated alkali lignin (SAL) at high temperatures. Lignin-based dye dispersants are environment-friendly, sustainable, and renewable.

The reduction of lignin-based dispersants poses obstacles for the application of azo dye, as azo disperse dyes degraded and lost color during the dyeing process [4]. Nevertheless, sulfonated lignin remains attractive due to its superior stability at high temperatures compared with naphthalene sulfonates [5].

**Figure 1:**
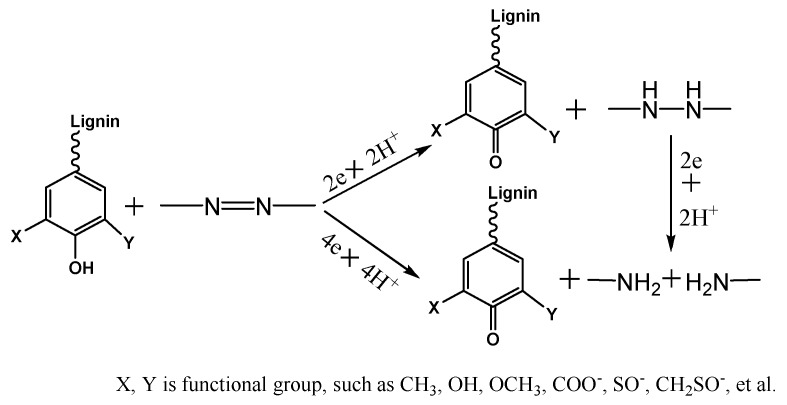
Reduction reaction for azo dye caused by lignin.

The azo dye reduction was inherent to the lignin structural functional group. Extensive studies showed that the easily oxidizable phenol hydroxyls in lignin were the primary cause for reduction [4]. The free phenol hydroxyls in lignin molecules could be easily oxidized to the quinoid structure, while the azo-type dye was converted to hydrazo and amine [5]. The redox reaction between lignin molecules and azo dyes [5,6] is illustrated in Figure 1.

The deep color of lignin caused by the quinoid structure further limited its application. An H_2_O_2_/UV radiation method [7] was attempted to brighten the lignin color; However, the reducing effect of lignin for azo dye remained. As phenolic groups are responsible for the azo dye reduction, blocking phenolic hydroxyl groups in lignin is an effective method to prevent azo dye reduction. Ultrafiltration [8] can remove small molecular lignin dispersants that often have a high hydroxyl group content [9]. Epoxy chloropropane [4], alkylene carbonate [10], and acetylation [11] were used to reduce the phenolic hydroxyl in the lignin molecule [12] but substantially decreased the lignin’s water solubility and dispersity without blocking the hydrophilic groups. 1,4-butane sultone (1,4–BS) was applied as a sulfonating and hydroxyl-blocking agent to produce a light-colored lignin-based dispersant from kraft lignin [13]. The prepared dispersant showed good dispersibility and remarkable stain resistance, but kraft lignin’s molecular weight was not increased and a lignin reducing effect was not reported.

Our previous studies have revealed that increasing the degree of sulfonation and molecular weight of lignin-based dispersants improved dispersity [14]. However, both sulfonation and polycondensation preferentially take place on the ortho position of the phenolic hydroxyl in AL molecules when preparing SAL [15]. The two competitive reactions in the sulfomethylating process resulted in a trade-off between increasing the sulfonation and molecular weight [16]. AL molecules have a high phenol hydroxyl content owing to depolymerization under highly alkaline and high-temperature conditions during the pulping process. The reduction problem was obvious when using SAL as the dye dispersant. Producing a lignin-based dye dispersant with a low reduction effect, high content of sulfonic groups, and high molecular weight remains a challenge.

_ENREF_11_ENREF_11In this work, a novel lignin-based dispersant was developed by a grafting sulfonation and blocking etherification reaction using alkali lignin as the raw material. The chemical structures and dispersive performance of GSBAL, AL, and NaLS were characterized by FT-IR, NMR, molecular weight, and SEM analysis. The reducing effect of these lignin dispersants for azo dye was investigated. Furthermore, the dye exhaustion was also determined to reveal the overall performance of modified lignin-based dye dispersants.

## 2. Materials and Methods

### 2.1. Materials

Alkali lignin (AL, wheat straw alkali lignin, from Tumen Papermaking Co. Ltd., Jilin, China), contains 90 wt% lignin, 6% sugars (include glucose, xylose, and arabinose), and 4% inorganic salts.

Sodium lignosulfonate (NaLS), obtained from Borregaard Co. Ltd. (Shanghai, China). It consists of 91% NaLS, 4.7% inorganic salts, and 4.3% sugars (include glucose, xylose, galactose, and arabinose).

The disperse dye, C.I. disperse blue 79 (one kind of azo dye, C_24_H_27_BrN_6_O_10_ [17], CAS registry number: 12239-34-8 3618-73-3, the purity is 98%) from Zhejiang Runtu Co., Ltd. (Hangzhou, China).

Polyester fiber came from RongSheng petrochemical Co. Ltd. (Hangzhou, China).

### 2.2. Preparation of Sulfomethylated Alkali Lignin (SAL)

**Figure 2:**
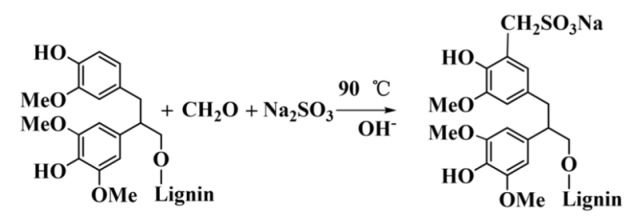
The reaction equation of the preparation of sulfomethylated alkali lignin (SAL).

AL (100 g) was dissolved in 300 mL of alkali solution with pH = 12 prepared using distilled water and a NaOH solution of concentration 2 mol·L^−1^. A mixed solution with 10 g of Na_2_SO_3_ and 10 g of formaldehyde was added into the AL solution. The mixture was heated to 90 °C and maintained for 2.5 h. Sulfomethylated alkali lignin (SAL) was obtained after cooling to room temperature. The reaction is shown in Figure 2.

### 2.3. Grafting Sulfonated and Blocking Condensed Alkali Lignin (GSBAL)

The reaction intermediate sodium 3-chloro-2-hydroxy-propanesulfonate was prepared according to Geng et al. [18]. The following were added to a flask with stirring: 20 g of NaHSO_3_, 70 mL of water, and 0.2 g of NaOH. Epichlorohydrin (16 g) was added slowly (within 30 min) at 20–25 °C. Then, the temperature was raised to 45 °C and maintained for 2 h.

**Figure 3:**
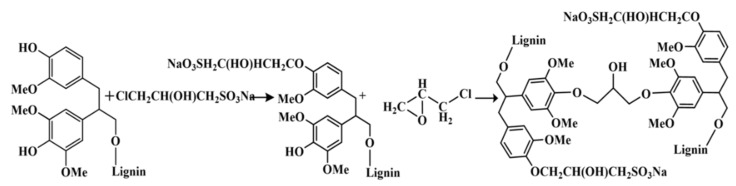
The reaction equation of the preparation of grafting sulfonated and blocking condensed alkali lignin (GSBAL).

AL (100 g) was solubilized into 100 mL of distilled water at pH 10 (adjusted with NaOH solution of 2 mol·L^−1^). The AL solution was heated to 90 °C. The prepared sodium 3-chloro-2-hydroxy-propanesulfonate was then added into the AL solution, and the reaction proceeded at 90 °C for 2 h. Afterward, a polymerization reaction was carried out by adding epichlorohydrin (8 g) dropwise and then maintained for 1 h. After cooling, the GSBAL was obtained. The reaction is shown in Figure 3. Sodium 3-chloro-2-hydroxy-propanesulfonate can also react with S PhOH or with G PhOH or with both S and G PhOH. Due to the complex structure of AL, Figure 3 illustrates a simplified ideal reaction model, in which Sodium 3-chloto-2-hydroxypropane sulfonate reacts solely with G PhOH.

### 2.4. FTIR Analysis

FTIR was carried out using an FTIR Spectrometer (Nicolet Nexus 470, Thermo Nicolet Corporation, Madison, WI, USA). The samples were prepared by mixing 2 mg of dried sample into 200 mg of KBr to form tablets.

### 2.5. HSQC Analysis

Al and GSBAL 2D-NMR (HSQC) spectra were recorded using a Bruker spectrometer (DRX-500, Bruker Co., Ettlingen, Germany). A quantity of 60 mg dried AL and GSBAL was dissolved into 0.6 mL of DMSO-d_6_ and 0.6 mL of D_2_O, respectively.

### 2.6. Molecular Weight Distribution Analysis

The molecular weight distribution was measured using Waters Styragel columns GPC equipment (Waters 2487 UV absorbance detector, Waters Crop., Milford, MA, USA). Sodium polystyrene sulfonate with different molecular weights was used as the standard for calibration. A NaNO_3_ solution of 0.1 mol·L^−1^ was used as the mobile phase. All samples were adjusted to 10 mg·L^−1^ using double-distilled water and filtered through a 0.22 μm syringe filter before testing.

### 2.7. Sulfonic Group Content Measurement

The sulfonic group content was determined by the potentiometric titration method using an automatic potentiometric titrator (809 Titrando, Metrohm Corporation, Herisau, Switzerland) [19]. Before titration, the samples were ion-exchanged through the anion exchange resin and cation exchange resin to remove salts and other impurities. A 0.05 mol·L^−1^ NaOH standard solution was used as the titrant. The first-order derivative peak of the titration curve at pH windows of 5.5–5.8 is the titration end point. The titration temperature was kept at 25 °C. The sulfonic group content can be calculated using Equation (1):

(1) $$\;\mathrm{Sulfonic}\;\mathrm{group}\;\mathrm{content}=\frac{mmole\;of\;SO_{3}H\;}{dried\;sample\;\left(g\right)}$$

### 2.8. Phenolic Hydroxyl Content Measurement

The phenolic hydroxyl content was measured using the Folin-Ciocalteu (FC)-reagent method [20]. First, dissolve 50 mg of dried dispersant into 100 mL water. Thoroughly mix 15 mL of the prepared sample solution with 1.5 mL of the FC-reagent. This is followed by adding 5 mL of 20% (*w*/*v*) Na_2_CO_3_ solution and adjusting the volume to 25 mL using distilled water. The final mixture was kept stirring for 2 h at 30 °C. A UV-Vis spectrophotometer (UV-2450, Shimadzu Co., Tokyo, Japan) was used to measure the absorption of the final mixture at 760 nm to determine the phenolic hydroxyl content. Vanillin solutions with different concentrations were used for calibration.

### 2.9. Preparation of Dye Suspension

A total of 7.5 g of dried dispersant and 5 g of dried dye filter cake were ball-milled using a planetary ball mill loaded with 200 g agate beads (QM-3SP2, Nanjing University Instrument Co., Nanjing, China). The solid content of the mixture was adjusted to 30 wt% and pH was maintained at 5.5 by adding HAc/NaAc buffer solution. The dye suspension was obtained by milling for 6 h at a rate of 400 r·min^−1^.

### 2.10. Dye Exhaustion Measurement

The polyester fibers were pretreated before dying. An acetate buffer solution of pH 5.0 was prepared by using 0.05 mol·L^−1^ of anhydrous sodium acetate and 0.0275 mol·L^−1^ of acetic acid. Fibers were washed four times using hot water at 95 °C and maintained for 45 min for each washing, and then soaked in the prepared buffer solution for 1 day before being dried for dyeing.

An intelligent controlled GRY-12 dyeing equipment (Quanrun Machinery Co. Ltd., Wuxi, China) was employed to carry out the dyeing process. The dye bath was obtained from the dye suspension (the preparation of which was described in Section 2.9) by diluting to 0.5 wt% using distilled water and adjusting the pH to 5.5 using an HAc/NaAc buffer solution. A total of 2.0 g of pretreated dried fibers were added into the 200 mL dye bath (liquid/solid ratio = 100:1). The dyeing temperature was first raised from 20 to 130 °C at 2 °C min^−1^ and maintained at 130 °C for 45 min followed by cooling to 80 °C at 4 °C·min^−1^.

A UV spectrometer (Shimadzu Corp., Tokyo, Japan) was used to determine the dye exhaustion by testing the absorbance of 0.02 g·L^−1^ of dye bath (diluted to with acetone) at 580 nm. The dye exhaustion was calculated using Equation (2).

(2) $$\;\varepsilon =\frac{A_{i}-A_{f}\;}{A_{i}}\times 100\%$$

where *ε* is the dye exhaustion, *A_i_* and *A_f_* are the absorbance of the dye bath without and with dyeing process, respectively.

### 2.11. Scanning Electron Microscope (SEM) Images Measurement

The prepared dye bath (200 mL) in Section 2.10 was placed into the dyeing vessel. The experiment process described in Section 2.10 was implemented without using polyester fiber. The SEM images of samples were recorded with SEM instrument (Zeiss Netherlands BV, Sliedrecht, The Netherlands).

### 2.12. Reducing Rate of Dispersant on Azo Dye

The reducing property of the dispersant on disperse blue 79 was measured at 25 and 130 °C respectively by a UV spectrophotometer (UV-2450, Shimadzu Corp., Tokyo, Japan). The temperature of the dye bath was kept at 25 and 130 °C for 45 min, respectively. Then, the dye bath was diluted to 0.02 g·L^−1^ with acetone to prepare a dyestuff composition. Disperse blue 79 (dye filter cake) was dissolved into acetone to prepare a dye solution of 0.02 g·L^−1^ not containing dispersant as the control sample at 25 and 130 °C. The azo percentage is calculated by Equation (3).

(3) $$\;\mathrm{Dye}\;\mathrm{reducing}\;\mathrm{rate}\;\%=\frac{A_{0}-A_{t}\;}{A_{0}}\;\times 100$$

where, *A*_0_ is absorbance of the dye in acetone at λ_580_, *A_t_* is the absorbance of a dye bath in acetone at λ_580_.

## 3. Results and Discussion

### 3.1. FTIR Analysis

**Figure 4:**
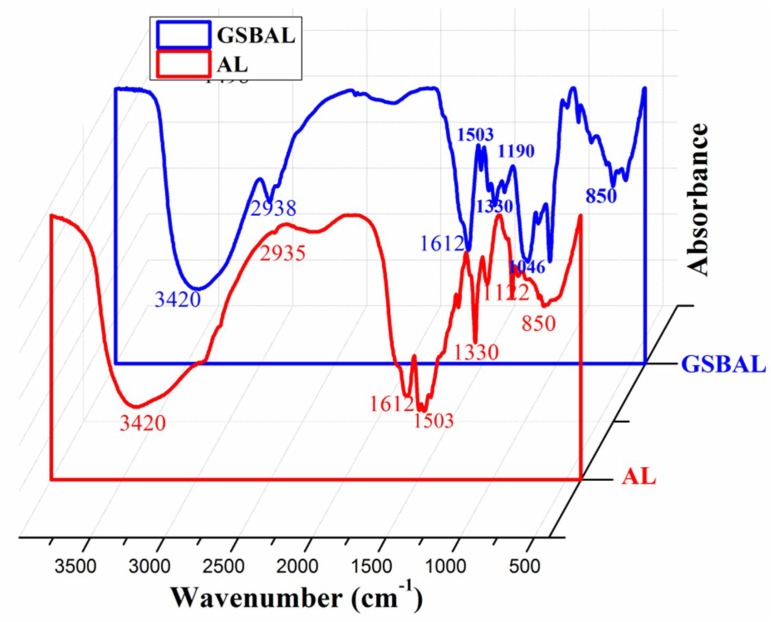
FTIR spectra of alkali lignin (AL) and GSBAL.

FTIR spectra of AL and GSBAL were recorded and represented in Figure 4. The semi-quantitative method of FTIR was carried out to reveal the hydroxy structural changes of lignin [21,22]. The areas of bands at 1503 cm^−1^ observed in both samples corresponding to aromatic ring stretching vibrations [22] were calculated. The reactions occurred on the phenolic hydroxyl group, which would not affect the aromatic ring structure of lignin [21,22]. The peak at 1503 cm^−1^ could be regarded as the reference peak. The band at 3420 cm^−1^ related to the aromatic and aliphatic OH group in lignin [23]. The ratio of the peak area at 3420 cm^−1^ and 1503 cm^−1^ (*A3420/A1503*) of AL and GSBAL was 61.32 and 23.46, respectively. *A3420/A1503* of GSBAL essentially decreased compared with AL, suggesting that most of the OH groups were blocked by epichlorohydrin. The peaks at 2935–2938 cm^−1^ assigned to the stretching vibration of CH_2_ or CH_3_ [24], became intense in GSBAL, which indicated that the hydroxy-propane sulfonate contained methyl and that the methylene group was grafted into lignin. The band at 1330 cm^−1^ is related to the syringyl ring breathing with C–O stretching vibration [25]. The peaks at 1192 cm^−1^ and 1048 cm^−1^ are attributed to the asymmetric and symmetric stretching vibration of the sulfonic group from GSBAL [26], respectively. The signal at 850 cm^−1^ can be assigned to a C–H out-of-plane deformation vibration at the 2, 3, and 6 positions of the benzene ring [27].

### 3.2. ^1^H–^13^C Correlation NMR Spectrum (HSQC) of AL and GSBAL

**Figure 5:**
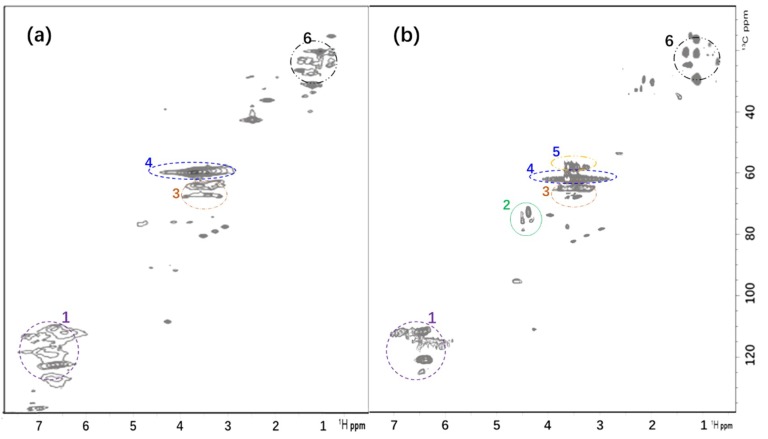
HSQC 2D-NMR analysis of (**a**) AL, reproduced with permission from [28]. Copyright Taylor & Francis 2013; and (**b**) GSBAL (signal assignment of marked numbers is shown in Table 1).

To further investigate the structure of AL and GSBAL, ^1^H–^13^C HSQC spectra of AL and GSBAL are shown in Figure 5, and the signal assignments are listed in Table 1. A wheat straw alkali lignin (AL) spectrum [28], which was recorded in our previous work, is shown as a comparison in Figure 5a.

The regions at 6.2–7.5/102–125 ppm of the GSBAL spectrum are attributed to aromatic H–C correlation. The region at 4.4–4.9/68–80 ppm is assigned to sulfonated side-chains H–C correlation. Meanwhile, the appearance of new signals at 3.0–3.6/53–57 ppm is attributed to methylene in the grafted hydroxypropyl sulfonate. This indicated that 3-chloro-2-hydroxy-propanesulfonate was successfully grafted into the AL molecule. The signal intensity at 3.0–4.4/56–62 ppm in both spectra is related to methoxy groups.

### 3.3. Molecular Weight and the Functional Group

The weight-average (*M_w_*), number-average (*M_n_*) molecular weights and the polydispersities (*M_w_/M_n_*) together with the contents of sulfonic group and phenolic hydroxyl group dispersants are listed in Table 2. The *M_w_* was increased from 4920 Da for AL to 11,350 Da for GSBAL. It indicates that epichlorohydrin reacts with phenolic hydroxyl groups of GSBAL and improves the *M_w_* more effectively than SAL. The *M_w_* of NaLS was 10,250 Da, which was higher than SAL. This is due to the removal of the low molecular weight fraction by ultrafiltration. Polydispersity, see Table 2, indicates the distribution of the polymer’s molecular weight. The polydispersity was decreased from 2.59 for AL to 1.93 for GSBAL, indicating that a blocking condensed process using epichlorohydrin increased the molecular weight with a more uniform distribution than SAL.

GSBAL had a higher sulfonic and a much lower phenolic hydroxyl content than those of NaLS and SAL. The phenolic hydroxyl group was 0.42 mmol·g^−1^, substantially lower than 2.27 mmol·g^−1^ of AL, suggesting that more than 80% of these were blocked. It can be inferred from the results that the copolymerization and grafting mainly took place on phenolic hydroxyl groups. SAL had low sulfonic and high phenolic hydroxyl contents, indicating that sulfonation was more competitive than polycondensation.

### 3.4. Dispersion Performance

**Figure 6:**
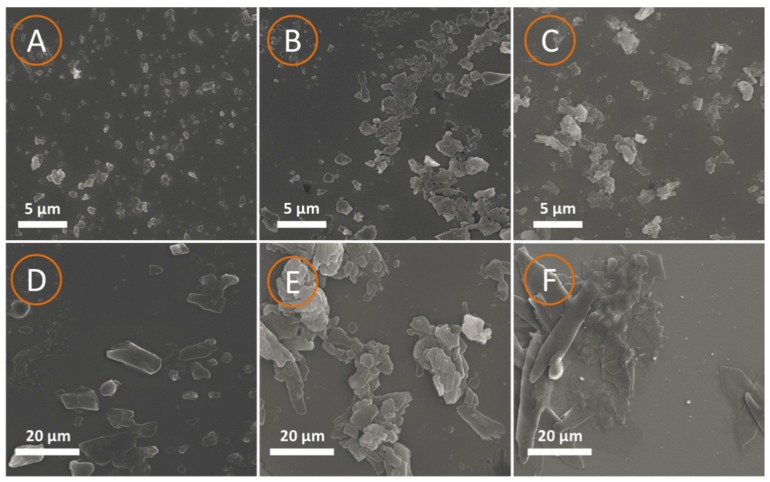
SEM images of dye bath with different dispersant (**A**) GSBAL at 25 °C; (**B**) sodium lignosulfonate (NaLS) at 25 °C; (**C**) sulfomethylated alkali lignin (SAL) at 25 °C; (**D**) GSBAL after 130 °C; (**E**) NaLS after 130 °C; (**F**) SAL after 130 °C.

The dye bath exhibited stability to high temperatures during dyeing to ensure excellent polyester fiber dyeing. Dye with smaller particle sizes in the dye bath presented better stability, and the dyeing efficiency was reported [33]. The morphology of the particles of the dye bath at 25 and 130 °C were measured and are shown in Figure 6.

The shape of the dye particles in a bath with GSBAL, NaLS, and SAL were all flake-like. At 25 °C, all three dispersants performed well in dispersing dye and grinding effects. However, different degrees of aggregation occurred at 130 °C using different dispersants, see Figure 6D–F. The size of the dye particles was only slightly increased to 9 μm using GSBAL. The particles of dye were substantially aggregated to approximately 36 and 42 μm, respectively, using NaLS and SAL. These results suggest that dye dispersed using GSBAL had better dispersibility and high-temperature stability than dye dispersed using NaLS and SAL.

GSBAL with higher molecular weight and a longer hydroxypropyl chain showed good dispersion by forming a stronger steric hindrance to keep the aqueous dye suspension stable under a high-temperature environment [14]. In the GSBAL molecule, phenolic hydroxyls were blocked while the aliphatic hydroxyl groups were introduced. Hydroxyl groups could be helpful by forming hydrogen bonds between a hydroxyl and the dye to stabilize dye particles in the dye bath [31]. This is an additional innovation of this work compared with other methods for reducing phenolic hydroxyls, such as oxidizing, and the acetylation process. Furthermore, the high degree of sulfonic groups in GSBAL using a graft sulfonation process also provided a strong electrostatic repulsion, increasing the stability of the dye bath [34].

### 3.5. Reducing Effect of Dispersant on Azo Dye

**Figure 7:**
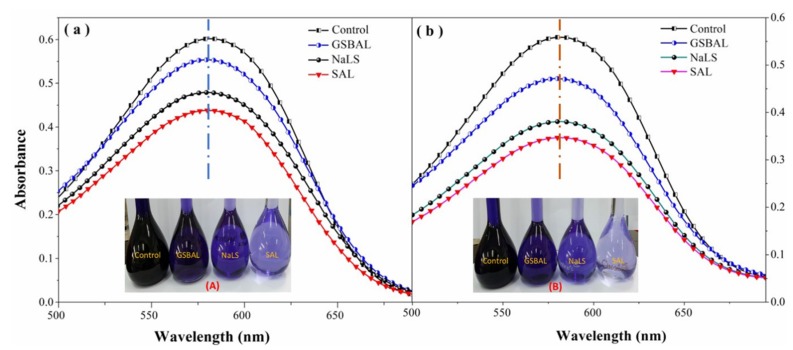
The absorbance of disperse dye baths with different dispersants: (**a**) 25 °C, (**b**) 130 °C; the dye concentration in each dye bath was 0.008 g·L^−1^, and dispersant (GSBAL, NaLS, and SAL) concentration was 0.012 g·L^−1^, respectively.

**Figure 8:**
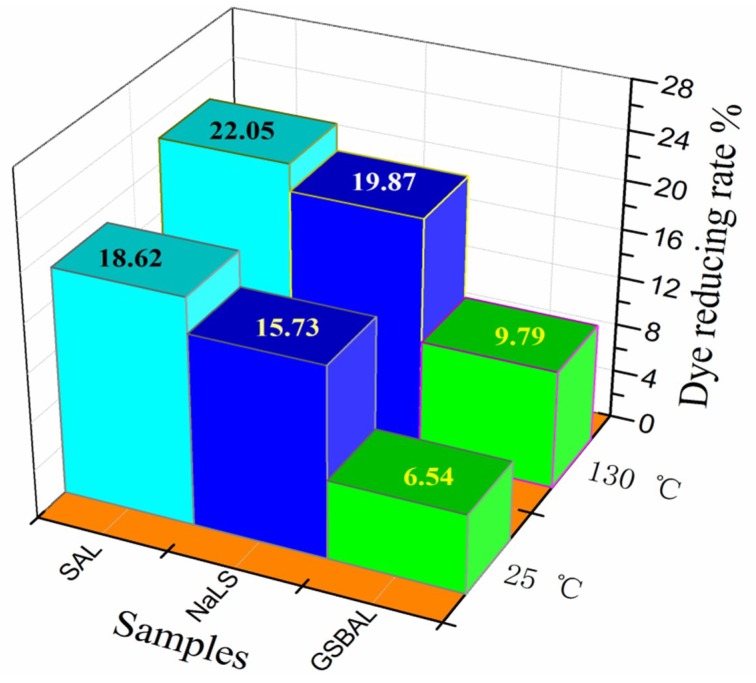
The reducing rate of dispersant at 25 °C and 130 °C.

The absorbances at 590 nm of the dye bath solutions using different dispersants are shown in Figure 7. The dye reducing rates are presented in Figure 8. The absorbance of the dye bath solution increased with the decrease of phenol hydroxyl groups in the dispersants. Dispersion using GSBAL achieved the highest absorption among all the dispersants with similar color to the control sample.

Although the dye reducing effect was aggravated by heating, the reducing rate of 9.79% of GSBAL (130 °C) was still much lower than 19.87% of NaLS and 22.05% of SAL. SAL had the highest reducing rate, ascribed to the sulfomethylated lignin, which had more phenolic hydroxyl groups than lignosulfonate [17].

### 3.6. Dye Exhaustion

**Figure 9:**
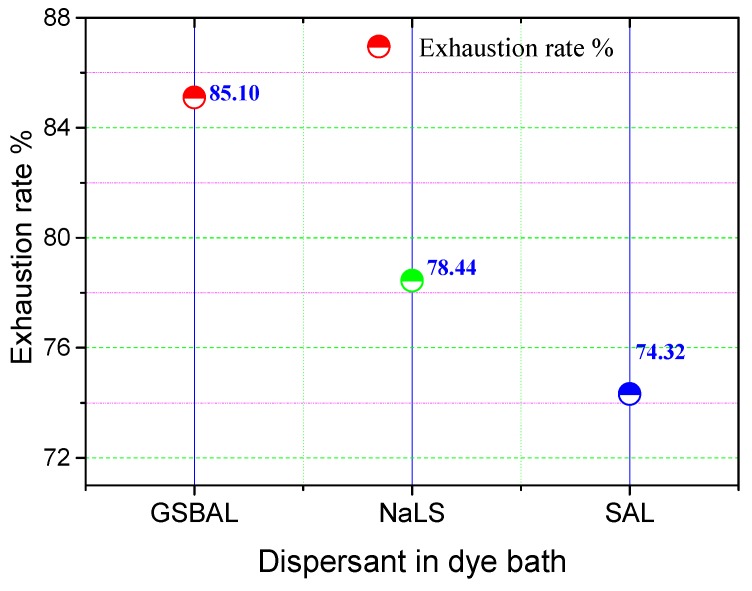
The exhaustion rate of dye baths with different dispersants.

The exhaustion results of disperse dye (C.I. disperse blue 79) are presented in Figure 9. The exhaustion rate of the dye bath with GSBAL reached the highest level of 85.10%, attributed to the higher content of effective dyes in the dye bath during heating as a result of the low reducing rate on disperse azo dye. The dyeing process of disperse dye consists of two steps [35]: The dissolution and redissolution of disperse dye adsorbed onto the fiber surface, followed by the diffusion of absorbed dye into the interior of fibers. The dye particles with GSBAL had better dispersion stability and high-temperature stability.

## 4. Conclusions

A novel lignin-based dye dispersant GSBAL was successfully prepared using alkali lignin by grafted-sulfonation with 3-chloro-2-hydroxy propyl sodium sulfonic acid and etherification with epichlorohydrin. The high sulfonic group content and high molecular weight of GSBAL with low phenolic hydroxyl group content exhibited good dispersity, and an ultra-weak reducing effect on disperse azo dye. Hence, GSBAL represented a high exhaustion.

## Figures and Tables

**Table 1:** Signal assignment of HSQC NMR spectra of AL and GSBAL.

Marked Number	δ_H_/δ_c_ ppm	Assignment
1	6.2–7.5/102–125	Aromatic ^1^H–^13^C [29]
2	4.4–4.9/68–80	^1^H–^13^C correlation in sulfonated chain (–CH_2_CH(OH)CH_2_SO_3_Na) [29,30]
3	3.4–3.7/62–72	Aliphatic oxygenated ^1^H–^13^C [31]
4	3.0–4.4/56–62	Methoxyl [32]
5	3.0–3.6/53–57	Methylene (part of sulfonated chain) [33]
6	0.5–1.5/15–30	Aliphatic non-oxygenated ^1^H–^13^C [31,32]

**Table 2:** Molecular weight data and functional groups of dispersants.

Dispersants	*M_w_* (Da)	*M_n_* (Da)	*M_w_*/*M_n_*	Sulfonic (mmol·g^−1^)	Phenolic Hydroxyl (mmol·g^−1^)
AL	4920	1900	2.59	—	2.27
NaLS	10,250	4780	2.14	1.36	1.88
SAL	6140	2200	2.79	1.14	2.16
GSBAL	11,350	5880	1.93	2.21	0.42

## References

[1] Zhao X., Hardin I.R., Hwang H.-M. (2006). Biodegradation of a model azo disperse dye by the white rot fungus Pleurotus ostreatus. Int. Biodeter. Biodegr..

[2] Koh J. (2011). Dyeing with Disperse Dyes, In Textile Dyeing.

[3] Huynh L., Beattie D.A., Fornasiero D., Ralston J. (2006). Effect of polyphosphate and naphthalene sulfonate formaldehyde condensate on the rheological properties of dewatered tailings and cemented paste backfill. Miner. Eng..

[4] Dilling P. (1984). Color Reduction Process for Non-sulfonated Lignin. U.S. Patent.

[5] Dilling P. (1986). Lignosulfonate dispersants and the role of the reduction of azo dyes. Text. Chem. Col..

[6] Liu L., Li F.B., Feng C.H., Li X.Z. (2009). Microbial fuel cell with an azo-dye-feeding cathode. Appl. Microbiol. Biot..

[7] Qiu X.Q., Yu J., Yang D.J., Wang J.Y., Mo W.J., Qian Y. (2017). Whitening sulfonated alkali lignin via H_2_O_2_/UV radiation and its application as dye dispersant. ACS Sustain. Chem. Eng..

[8] Dilling P., Samaranayake G.S. (1999). Mixtures of Amine Modified Lignin with Sulfonated Lignin for Disperse Dye. U.S. Patent.

[9] Yang D.J., Qiu X.Q., Pang Y.X., Zhou M.S. (2008). Physicochemical properties of calcium lignosulfonate with different molecular weights as dispersant in aqueous suspension. J. Dispers. Sci. Technol..

[10] Lin S.Y. (1980). Process for Reduction of Lignin Color. U.S. Patent.

[11] Qian Y., Zhong X., Li Y., Qiu X.Q. (2017). Fabrication of uniform lignin colloidal spheres for developing natural broad-spectrum sunscreens with high sun protection factor. Ind. Crops Prod..

[12] Pu Y., Ragauskas A.J. (2005). Structural analysis of acetylated hardwood lignins and their photoyellowing properties. Can. J. Chem..

[13] Zhang H., Yu B., Zhou W., Liu X., Chen F. (2018). High-value utilization of eucalyptus kraft lignin: Preparation and characterization as efficient dye dispersant. Int. J. Biol. Macromol..

[14] Qin Y.L., Yang D.J., Guo W.Y., Qiu X.Q. (2015). Investigation of grafted sulfonated alkali lignin polymer as dispersant in coal-water slurry. J. Ind. Eng. Chem..

[15] Yang D.J., Qiu X.Q., Zhou M.S., Lou H.M. (2007). Properties of sodium lignosulfonate as dispersant of coal water slurry. Energ. Convers. Manag..

[16] Pang Y.X., Qiu X.Q., Yang D.J., Lou H.M. (2008). Influence of oxidation, hydroxymethylation and sulfomethylation on the physicochemical properties of calcium lignosulfonate. Colloids Surf. A.

[17] Park K.M., Yoon I., Lee S.S., Choi G., Lee J.S. (2002). X-ray crystal structure of CI Disperse Blue 79. Dyes Pigments.

[18] Geng X.F., Hu X.Q., Xia J.J., Jia X.C. (2013). Synthesis and surface activities of a novel di-hydroxyl-sulfate-betaine-type zwitterionic gemini surfactants. Appl. Surf. Sci..

[19] Lou H.M., Lai H.R., Wang M.X., Pang Y.X., Yang D.J., Qiu X.Q., Wang B., Zhang H.B. (2013). Preparation of lignin-based superplasticizer by graft sulfonation and investigation of the dispersive performance and mechanism in a cementitious system. Ind. Eng. Chem. Res..

[20] Sousa F.D., Reimann A., Jansson M.B., Nilberbrant N. Estimating the amount of phenolic hydroxyl groups in lignins. Proceedings of the 11th ISWPC.

[21] Zhou H., Yang D., Qiu X., Wu X., Li Y. (2013). A novel and efficient polymerization of lignosulfonates by horseradish peroxidase/H_2_O_2_ incubation. Appl. Microbiol. Biot..

[22] Tejado A., Pena C., Labidi J., Echeverria J., Mondragon I. (2007). Physico-chemical characterization of lignins from different sources for use in phenol–formaldehyde resin synthesis. Bioresour. Technol..

[23] Faix O., Lin S.Y., Dence C.W. (1992). Fourier transform infrared spectroscopy. Methods in Lignin Chemistry.

[24] Song Y., Wang Z., Yan N., Zhang R., Li J. (2016). Demethylation of wheat straw alkali lignin for application in phenol formaldehyde adhesives. Polymers.

[25] Jahan M.S., Chowdhury D., Islam M.K., Moeiz S. (2007). Characterization of lignin isolated from some nonwood available in Bangladesh. Bioresour. Technol..

[26] Bu L., Xing Y., Yu H., Gao Y., Jiang J. (2012). Comparative study of sulfite pretreatments for robust enzymatic saccharification of corn cob residue. Biotechnol. Biofuels.

[27] Hage R.E., Brosse N., Chrusciel L., Sanchez C., Sannigrahi P., Ragauskas A. (2009). Characterization of milled wood lignin and ethanol organosolv lignin from miscanthus. Polym. Degrad. Stabil..

[28] .Gan L., Zhou M., Yang D., Qiu X. (2013). Preparation and Evaluation of Carboxymethylated Lignin as Dispersant for Aqueous Graphite Suspension Using Turbiscan Lab Analyzer. J. Dispers. Sci. Technol..

[29] Hu T. (2009). Characterization of Lignocellulosic Materials.

[30] Lutnaes B.F., Myrvold B.O., Lauten R.A., Endeshaw M.M. (2008). ^1^H and ^13^C NMR data of benzylsulfonic acids-model compounds for lignosulfonate. Magn. Reson. Chem..

[31] Sakkayawong N., Thiravetyan P., Nakbanpote W. (2005). Adsorption mechanism of synthetic reactive dye wastewater by chitosan. J. Colloid Interf. Sci..

[32] Zhang L., Gellerstedt G. (2007). Quantitative 2D HSQC NMR determination of polymer structures by selecting suitable internal standard references. Magn. Reson. Chem..

[33] Willerich I., Li Y., Grohn F. (2010). Influencing Particle Size and Stability of Ionic Dendrimer Dye Assemblies. J. Phys. Chem. B.

[34] Kissa E. (1990). Partitioning and stability of aqueous dispersions. Effect of electrolytes on the stability of aqueous dye dispersions. Langmuir.

[35] Ujhelyiova A., Bolhova E., Oravkinova J., Tiňo R., Marcinčin A. (2007). Kinetics of dyeing process of blend polypropylene/polyester fibres with disperse dye. Dyes Pigments.

